# The Inhibitor of Growth Protein 5 (ING5) Depends on INCA1 as a Co-Factor for Its Antiproliferative Effects

**DOI:** 10.1371/journal.pone.0021505

**Published:** 2011-07-05

**Authors:** Feng Zhang, Nicole Bäumer, Miriam Rode, Ping Ji, Tao Zhang, Wolfgang E. Berdel, Carsten Müller-Tidow

**Affiliations:** 1 Department of Medicine, Hematology/Oncology, University of Münster, Münster, Germany; 2 Department of Pharmacology, School of Pharmacy, the Fourth Military Medical University, Xi'an, China; 3 Department of Pathology, MD Anderson Cancer Center, Texas University, Houston, Texas, United States of America; 4 Department of Thoracic Surgery, Tangdu Hospital, the Fourth Military Medical University, Xi'an, China; University of Texas MD Anderson Cancer Center, United States of America

## Abstract

The proteins of the Inhibitor of Growth (ING) family are involved in multiple cellular functions such as cell cycle regulation, apoptosis, and chromatin remodeling. For ING5, its actual role in growth suppression and the necessary partners are not known. In a yeast-two-hybrid approach with human bone marrow derived cDNA, we identified ING5 as well as several other proteins as interaction partners of Inhibitor of cyclin A1 (INCA1) that we previously characterized as a novel interaction partner of cyclin A1/CDK2. ING5 expression in leukemic AML blasts was severely reduced compared to normal bone marrow. In line, ING5 inhibited bone marrow colony formation upon retroviral transduction. However, *Inca1* ^−/−^ bone marrow colony formation was not suppressed by ING5. In murine embryonic fibroblast (MEF) cells from *Inca1^+/+^* and *Inca1^−/−^* mice, overexpression of ING5 suppressed cell proliferation only in the presence of INCA1, while ING5 had no effect in *Inca1^−/−^* MEFs. ING5 overexpression induced a delay in S-phase progression, which required INCA1. Finally, ING5 overexpression enhanced Fas-induced apoptosis in *Inca1^+/+^* MEFs, while *Inca1^−/−^* MEFs were protected from Fas antibody-induced apoptosis. Taken together, these results indicate that ING5 is a growth suppressor with suppressed expression in AML whose functions depend on its interaction with INCA1.

## Introduction

Several ING tumor-suppressor family proteins (ING1-5) have been discovered during the past decade. The founding member of the family, ING1, was first identified by subtractive hybridization between normal human cells and breast cancer cell lines and was found to be suppressed in cancer cells [1]. Subsequently, ING1 was demonstrated to cooperate with p53 to induce apoptosis and cellular senescence [2], [3]. Since the discovery of ING1, four additional genes (ING2-5) [4], [5], [6], [7] have been identified and classified as ING family. All ING proteins share a highly conserved carboxy-terminal plant homeodomain (PHD) and are involved in cell cycle regulation, apoptosis and DNA repair [8], [9]. Studies have shown that ING proteins exert their biological function through their association with specific molecular partners [10], [11], [12].

The cell cycle is tightly regulated by different cyclin-CDK complexes [13], [14], [15]. An alternative cyclin A, named cyclin A1 [16], associates with CDK2 and is involved in mitosis, meiosis and malignant diseases [17], [18], [19], [20]. Cyclin A1 is highly expressed in Acute Myeloid Leukemia (AML) and cyclinA1 overexpression can induce leukemia. However, the detailed molecular functions of cyclin A1 remain unclear. In a study aimed to identify interaction partners and substrates of cyclin A1, INCA1, a novel protein, was found in a yeast triple-hybrid system to interact with cyclin A1/CDK2 complex [21]. First functional analyses indicate a growth-suppressive function through inhibition of CDK2 activity by INCA1 [21]. Recently, we generated an *Inca1* knockout mouse model to further study the *in vivo* function of this new protein [22] (manuscript in preparation). In the present study, by a yeast two-hybrid approach, we identified several potential interacting proteins of INCA1 from a bone marrow cDNA library. We confirmed nine interacting proteins with INCA1 by GST pull-down assay. ING5 was identified as one of the interacting partners of INCA1.

ING5 is the new member of ING family which was identified by computational homology search. Up to now, there are not many published data about ING5 functions. ING5 has been shown to physically interact with p300 and p53 *in vivo*, and ING5 overexpression induces apoptosis in colorectal cancer cells [7]. Recent study finds mutation and downregulation of ING5 mRNA in oral squamous cell carcinoma, suggesting it as a tumor suppressor gene [23]. Data from tissue array showed that ING5 translocation from the nucleus to the cytoplasm might be a critical event for carcinogenesis and tumor progression in human head and neck squamous cell carcinoma [24], [25]. In addition, aberrant ING5 expression was thought to contribute to pathogenesis, growth, and invasion of gastric carcinomas and colorectal cancer [26], [27]. The conflicting views of ING5 as a tumor suppressor or an oncogene are clearly context specific.

We found a significant reduction of ING5 expression in AML patients, which supports a function of ING5 as a tumor suppressor. We then focused our study on the interaction of ING5 and INCA1 and the consequence on cell proliferation, cell cycle and apoptosis. Our results indicate a close dependence of ING5 on the presence of INCA1 for the regulation of colony formation, cell proliferation, and apoptosis.

## Results

### Identification of interacting partners of INCA1

**Figure 1:**
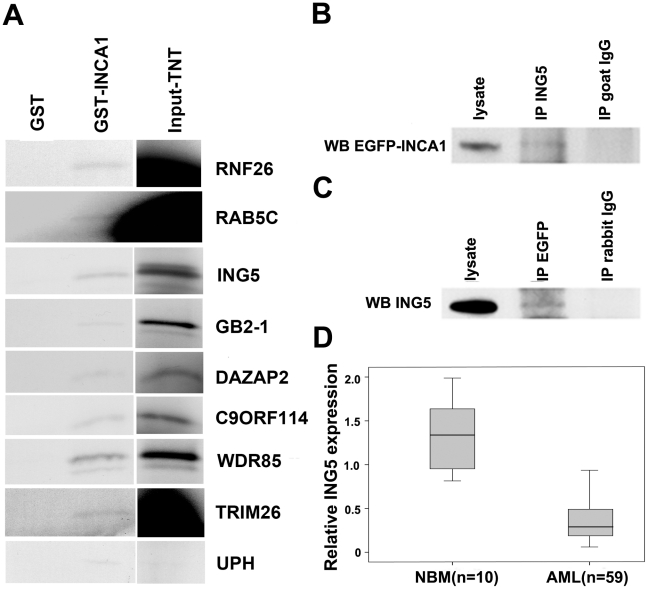
ING5 interacts with INCA1 *in vitro* and *in vivo*. (A) In GST pull-down assays, GST alone or GST fused to INCA1, were incubated with [^35^S] labeled genes which were selected through a yeast two-hybrid (Y2H) screening. Nine known genes were confirmed to interact with INCA1 *in vitro*. (B) COS-7 cells were transfected with EGFP-INCA1 and ING5. Immunoprecipitation with anti-EGFP antibody and subsequent Western blotting for ING5 demonstrated the *in vivo* interaction of ING5 and INCA1. (C) Immunoprecipitation with anti-ING5 antibody and subsequent Western blotting for EGFP-INCA1 confirmed the *in vivo* interaction. (D) Ing5 gene expression was decreased in AML specimens as determined by quantitative real-time RT-PCR assays based on Taqman technology. AML specimens were obtained at the time of diagnosis (p = 0.02).

To further address the molecular function of INCA1, we screened a human bone marrow cDNA library for interacting proteins of INCA1. Positive clones were confirmed by colony-lift filter assay and β-galactosidase activity. Sequences from 245 positive clones were analyzed by alignment to the NCBI data bases and 30 genes from the positive clones were selected for further investigation. We first confirmed the in vitro interactions by GST pull-down assay using *in vitro* transcribed and translated proteins. As described previously [21], the full length INCA1 cDNA was cloned, expressed as GST fusion proteins in *Escherichia coli* and purified using glutathione-agarose beads. INCA1 GST protein was incubated with *in vitro* transcribed and translated TNT system labeled with [^35^S] methionine. Nine known genes interacted with GST-INCA1(Fig. 1A, Table 1), but not with GST alone, indicating the specific interactions with INCA1 *in vitro*.

**Table 1:** Genes identified in a yeast two-hybrid system for INCA1 interaction partners.

	Gene	Accession No.
1	DAZ associated protein 2 (DAZAP2)	BC002334
2	Inhibitor of growth family, member 5 (ING5)	NM_032329
3	Ring finger protein 26 (RNF26)	BC007534
4	Guanine nucleotide binding protein (G protein), beta polypeptide 2-like 1	BC032006
5	Ubiquitin-specific protease homolog (UPH)	AF153604
6	Chromosome 9 open reading frame 114(C9orf114)	BC046133
7	WD repeat domain 85 (WDR85)	NM_138778
8	Tripartite motif-containing 26 (TRIM26)	BC032297
9	RAB5C, member RAS oncogene family	BT019484

### ING5 interacts with INCA1 *in vitro* and *in vivo*

Among the nine interacting partners of INCA1, ING5 is a new member of the candidate tumor-suppressor ING family. Since cyclin A1 can act as an oncogene, we further focused on the functions of the interaction between INCA1 and ING5. To further investigate this interaction *in vivo*, COS-7 cells were transfected with expression plasmids for EGFP-INCA1 and ING5. ING5 was immunoprecipitated from whole cell lysates with ING5 antibody. The subsequent Western blotting for EGFP-INCA1 demonstrated the specific interaction of INCA1 and ING5 *in vivo* (Fig. 1B). INCA1 was not precipitated from the cell lysates by nonspecific antibodies (Fig. 1B). Similarly, ING5 was also precipitated form the cell lysates by anti-EGFP antibody (Fig. 1C). These data indicated a direct interaction between INCA1 and ING5 in vitro and at least upon overexpression also in vivo. Due to the absence of good quality antibodies for INCA1, no CO-IPs at normal levels in vivo could be performed.

### ING5 expression is suppressed in AML patients and ING5 overexpression decreases colony formation efficiency in the presence of INCA1

We further analyzed Ing5 gene expression at the mRNA level in AML specimens obtained at the time of primary diagnosis. These analyses revealed that Ing5 was expressed at significantly lower levels in AML compared to normal bone marrow (Fig. 1D, *P* = 0.02), hinting at a potential growth suppressive function of ING5. We then focused our study onto the functional interactions of ING5 and INCA1 on cell growth control.

**Figure 2:**
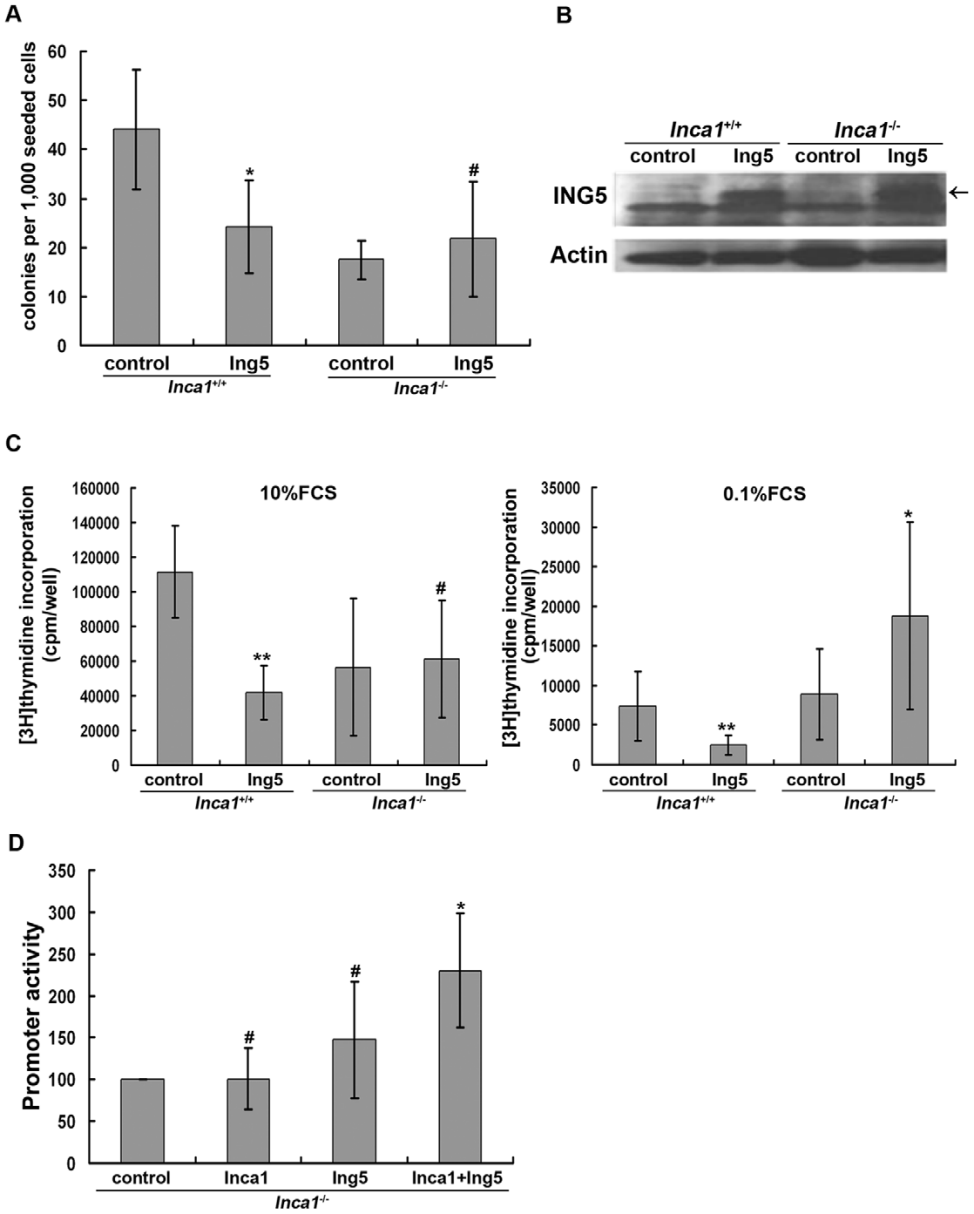
Inhibition of cell growth by ING5 depends on INCA1. (A) *Inca1* ^+/+^ and *Inca1* ^−/−^ bone marrow cells were retrovirally transduced with ING5 or empty vector, sorted by FACS, and then subjected to colony formation assays. Data are shown as mean plus standard error of three independent experiments (*P<0.05 compared to *Inca1* ^+/+^ control; ^#^not significant). (B) Immortalized MEF cells from *Inca1* ^+/+^ and *Inca1* ^−/−^ were retrovirally transduced with ING5 or empty vector. After being sorted by FACS, four stably transduced cell lines were established as bulk culture. ING5 overexpression was confirmed by Western blotting with anti-ING5 antibody. (C) Cell lines were cultured in medium containing 10% FCS or 0.1% FCS and analyzed for proliferation using [^3^H]thymidine incorporation. Data are shown as mean plus standard error of three independent experiments (**P<0.01 compared to *Inca1* ^+/+^ control; ^#^not significant, *P<0.05 compared to *Inca1* ^−/−^ control). (D) *Inca1* ^−/−^ MEFs were transfected with ING5, INCA1 or both, and activation of a p53-responsive promoter was analyzed by luciferase assay. ING5 and INCA1 co-transfection led to activation of the promoter. Data are shown as mean plus standard error of three independent experiments (^#^not significant, *P<0.05 compared to control).

We first performed colony formation assay with primary bone marrow cells. ING5 expressing or empty vector were retrovirally transduced into lineage depleted bone marrow cells obtained from *Inca1^+/+^* and *Inca1^−/−^* mice followed by FACS sorting for EGFP positive cells. Colonies were counted after one week of culture. ING5 overexpression significantly inhibited colony formation in primary wildtype bone marrow. This is consistent with previous reports in other cell types that overexpression of ING5 in cancer cells resulted in reduced colony formation [7]. As a surprise, ING5 overexpression did not inhibit colony formation of *Inca1^−/−^* bone marrow cells (Fig. 2A). These data indicated that ING5 overexpression could decrease colony formation only in the presence of INCA1.

### The anti-proliferative effects of ING5 depend on INCA1

Immortalized MEFs derived from *Inca1^+/+^* and *Inca1^−/−^* mice were retrovirally transduced with empty vector (“control”) or ING5. Four cell lines (*Inca1^+/+^* control, *Inca1^+/+^* Ing5, *Inca1^−/−^* control and *Inca1^−/−^* Ing5) were established after sorting for GFP positive cells by FACS. ING5 overexpression was confirmed by Western blot (Fig. 2B).

To determine the effect of ING5 overexpression on cell proliferation, [^3^H]-thymidine incorporation assays were performed. When cultured in medium containing 10% FCS or 0.1% FCS, overexpression of ING5 in *Inca1^+/+^* MEFs inhibited cell proliferation compared to wildtype control cells (*P*<0.01; Fig. 2C). Overexpressed ING5 did not inhibit the proliferation of *Inca1^−/−^* MEFs (Fig. 2C). In *Inca1^−/−^* MEFs cultured in 0.1% FCS medium, ING5 overexpression rather increased proliferation (*P*<0.05) compared to *Inca1^−/−^* control cells (Fig. 2C, righthand panel), showing a similar tendency as observed in colony formation assay. Taken together, these results indicated that the growth inhibitory effects of ING5 required INCA1.

### INCA1 is important for the activation of p53-responsive promoter by ING5

ING5 overexpression inhibited cell growth in a p53-dependent manner by increasing *p21/waf1* promoter activity [7]. We analyzed the effects of INCA1 on the promoter activation function of ING5. Therefore, we transfected *Inca1^−/−^* MEFs with expression vectors containing ING5, INCA1 or both, and we analyzed the activity of a p53-responsive promoter. ING5 overexpression modestly increased the activity of the p53-responsive promoter with no statistical significance whereas INCA1 overexpression had no effect (Fig. 2D). However, when ING5 and INCA1 were co-transfected, promoter activity increased about 2-fold (*P*<0.05).

### INCA1 plays an important role in the regulation of DNA replication by ING5

**Figure 3:**
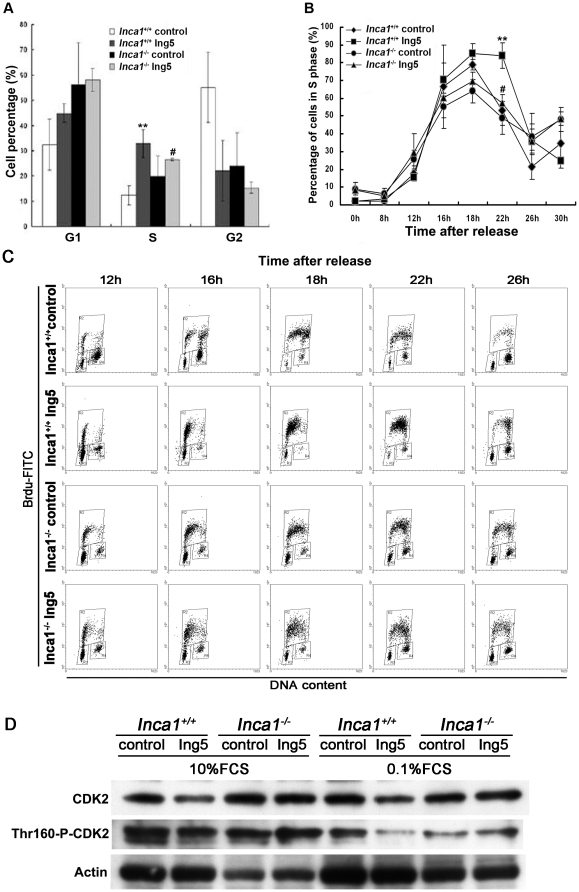
ING5 delays S-phase progression in an INCA1 dependent manner. (A) BrdU was incorporated to S-phase cell cultures by adding to the medium 1 h before harvesting. ING5 overexpression accumulated cells in S-phase only in the presence of INCA1. Data are shown as mean plus standard error of three independent experiments (**P<0.01 compared to *Inca1* ^+/+^ control; ^#^ not significant compared to *Inca1* ^−/−^ control). (B) Cells were starved for 48 h in 0.1% FCS and released into medium containing 10% FCS. Cells were collected at different time points after releasing for S-phase progression analysis. Representative figures of BrdU-FITC and PI staining showed that ING5 overexpression in *Inca1* ^+/+^ MEFs has prolonged S-phase and higher DNA synthesis activity. (C) Line figure clearly revealed the prolonged S-phase in *Inca1* ^+/+^ MEFs overexpressing ING5 compared to the other three cell lines. Data are shown as mean plus standard error of three independent experiments (**P<0.01 compared to the other three cell lines; ^#^ *P*>0.05 compared to *Inca1* ^−/−^ control). (D) The expression of both CDK2 and its active form (phosphor-Thr160-CDK2) was detected by western blotting in the four cell lines cultured under normal condition or 0.1% FCS culture medium for 48 h.

Since both ING5 and INCA1 were involved in cell cycle regulation [7], [10], [21], we analyzed cell cycle progression with PI staining by FACS. We first investigated how this interaction influenced cell cycle distribution in non-synchronized cell lines. In *Inca1^+/+^* MEFs with ING5 overexpression, there was an accumulation of cells in S-phase compared to *Inca1^+/+^* control MEFs (*P*<0.01; Fig. 3A), which was not significant in *Inca1^−/−^* MEFs (*P*>0.05; Fig. 3A).

To determine whether S-phase progression was affected, we focused specifically on the S-phase population using synchronized cells by starvation for 48 h in 0.1% FCS and release into medium containing 10% FCS. We used pulsed-labeling with BrdU to selectively label cells in S-phase. Cells were collected at the indicated time points after release. The S-phase fraction started to increase in all four cell lines 8 h after refeeding (Fig. 3B). In wildtype MEFs with ING5 overexpression, S-phase accumulation reached its peak at 22 h and decreased to the lowest level at 30 h. In all other cell lines, S-phase peaked at 18 h and began to decrease to the lowest points at 26 h (*P*<0.01). As shown in Figure 3C, ING5 overexpression in wildtype MEFs induced a marked delay in progression through S-phase for BrdU-labeled cells, and the inhibition occurred mainly in early S-phase. These data indicate that ING5 slows down S-phase progression in an INCA1-dependent manner.

CDK2 is an important regulator of S-phase progression. We detected expression of CDK2 in the four cell lines cultured under normal condition or 0.1% FCS culture medium for 48 h. Since activation of CDK2 complexes requires phosphorylation of Thr160, the level of its active form phospho-Thr160 CDK2 was analyzed. ING5 overexpression decreased both CDK2 expression and activity in wildtype MEFs compared to control MEFs especially at low serum conditions (Fig. 3D). Remarkably, expression or phophorylation of CDK2 could not be altered by ING5 in absence of INCA1 at both serum conditions (Fig. 3D).

Taken together, these results showed that overexpression of ING5 delays S-phase progression and accumulates cells in early S-phase, with downregulation of CDK2 activity. Notably, these effects are dependent on the presence of INCA1.

### ING5 increases anti-Fas antibody-induced apoptosis in an INCA1-dependent way

**Figure 4:**
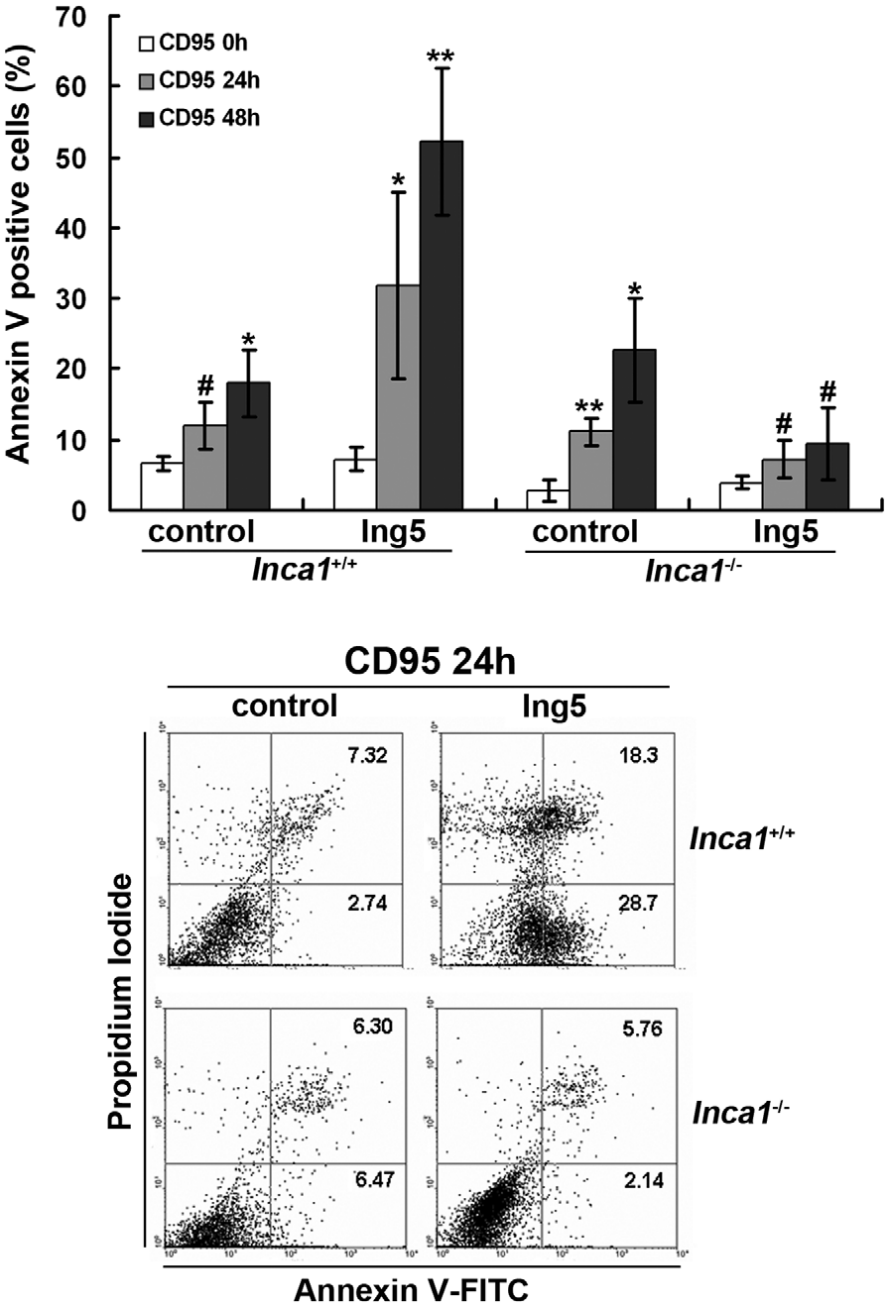
ING5 enhanced FAS antibody-induced apoptosis in an INCA1-dependent manner. MEF cell lines were exposed to anti-Fas antibody (1 µg/ml) for 0 h, 24 h or 48 h. Apoptosis was detected by AnnexinV-PI staining. Overexpression of ING5 in wildtype MEF increased apoptosis after 24 h and 48 h. However, in *Inca* ^−/−^ MEFs, ING5 overexpression prevented CD95-induced apoptosis. Data are shown as mean plus standard error of three independent experiments (*P<0.05, **P<0.01, and ^#^ *P*>0.05 compared to CD95 0 h with each cell line). A representative figure is shown in the lower panel.

It has previously been reported that ING5 overexpression results in increased apoptosis [7]. Overexpression of ING5, INCA1 or both in MEFs did not induce significant apoptosis (data not shown). Therefore, we examined whether the interaction between ING5 and INCA1 affected the sensitivity of cells to apoptosis-inducing agents such as TNFα, TRAIL and anti-Fas antibody. TNFα (200 ng/ml) and TRAIL (100 ng/ml) failed to induce apoptosis in all 4 MEF cell lines described above (data not shown), while apoptosis was detected when cells were exposed to Fas antibody (1 µg/ml) for 24 h or 48 h. Overexpression of ING5 in wildtype MEFs caused a dramatically increased apoptotic rates at 24 h (*P*<0.05) or 48 h (*P*<0.01) compared to *Inca1^+/+^* control MEFs (Fig. 4). In *Inca1^−/−^* MEFs, Fas antibody treatment for 24 h or 48 h only induced apoptosis in control vector cells, but not in ING5 overexpressing cells, suggesting that ING5 overexpression renders MEFs resistant to Fas-induced apoptosis in the absence of INCA1.

To investigate whether Fas expression was affected, we determined expression of membrane Fas and total Fas by flow cytometry. ING5 overexpression down-regulated both membrane and total Fas expression in *Inca1^−/−^* and *Inca1^+/+^* MEFs (Figure S1), which is not consistent with the results of apoptosis, suggesting that other mechanisms may mediate the process. These results also indicate that INCA1 plays a key role in this pathological process for determining cell fate, which is in line with the results we obtained from colony formation and proliferation assays.

## Discussion

INCA1 was found in a yeast triple-hybrid system as a novel protein to interact with cyclin A1/CDK2 complex [21]. In the present study, we used a yeast two-hybrid screen system to identify the interaction partners of INCA1 in bone marrow. The interaction was verified for all isolated proteins by GST pull-down assays. The interacting proteins included eight known proteins (DAZAP2, ING5, RNF26, G protein, UPH, WDR85, TRIM26 and RAB5C) as well as one protein with unknown function (C9orf114). Among the interacting proteins, ING5, a new member of Inhibitor of Growth family, was functionally analyzed. ING5 interacted with INCA1 and was down-regulated in AML blast cells. We therefore chose ING5 and studied the interaction between INCA1 and ING5 and its effects on cell growth control including colony formation, cell proliferation, cell cycle progression, and apoptosis.

It has been reported that overexpression of ING5 in cancer cells resulted in reduced colony formation efficiency through interacting with P53 and P300 [7]. In our study, we also found that ING5 overexpression could inhibit colony formation of mouse bone marrow cells, however, this effect existed only in the presence of INCA1. In *Inca1^−/−^* MEFs, this inhibitory effect of ING5 was completely abolished. These results suggest that the interacting partners of ING5 are important for its functions, and INCA1 is indispensable for the growth inhibition effect of ING5.

P300 is a member of histone acetyl transferase complexes. By interacting with P53 and P300, ING5 overexpression could enhance p53 acetylation at Lys-382 residues, which is involved in transcriptional activity of p53 [7]. We also observed in *Inca1^−/−^* MEF cells, that co-transfection of both ING5 and INCA1 increased the activity of a p53-responsive promoter, while ING5 alone increased the promoter activity to a much less extent with no statistical significance. These data confirmed the results of Harris's group [7], though they were obtained from different cell types. In addition, our results suggest that the p53 transcriptional activation effect of ING5 is partly dependent on its interaction with INCA1.

Recent study suggest that ING family proteins may play dual roles, as tumor suppressors or oncogenes, under different cellular conditions [28]. The current study of proliferation assay with MEFs revealed that ING5 overexpression inhibited cell proliferation only in *Inca^+/+^* MEFs, but not in *Inca1^−/−^* MEFs, indicating that ING5 inhibits cell growth in an INCA1-dependent manner. Unexpectedly, when *Inca1^−/−^* MEFs were cultured in 0.1% FCS medium, ING5 overexpression even significantly increased cell proliferation, showing an oncogenic property. These results suggest that the two facets of ING5 functions in MEFs could be affected by INCA1 interaction. It could be speculated that by interacting with different protein partners, ING5 may function as a tumor suppressor or an oncogene.

ING5 plays a similar role in apoptosis assay. It has been observed that ING5 overexpression in cancer cells results in increased apoptosis, which is p53-dependent [7]. Here we show that ING5 overexpression enhances anti-Fas antibody triggered-apoptosis, which is totally dependent on INCA1 presence. INCA1 absence even reverses the apoptosis-enhancing effect of ING5 to apoptosis resistance. These data further confirm that the growth suppressive function of ING5 relies on its interaction with INCA1.

By measuring BrdU incorporation, Doyon *et al.* [10] showed that ING5 complexes with histone acetyltransferases are important for DNA replication. ING5 knockdown cells almost completely lack DNA synthesis, suggesting an essential role of ING5 in DNA replication. Our results confirmed that ING5 overexpression increased DNA synthesis and accumulated cells in S-phase. In addition, ING5 also affects cell cycle progression, especially S phase. In starvation-synchronized wildtype MEFs ING5 overexpression caused a delay in early S-phase. ING5 overexpression also decreased CDK2 activity only in the presence of INCA1 in cells cultured in normal medium or 0.1% FCS medium. Based on these results, it is possible that in addition to promoting replication, ING5 might play a role in the intra-S-phase checkpoint activation which may slow the rate of DNA replication [29]. The intra-S checkpoint is active in normal S-phase, which means some replication stress occurs in a normal S-phase, not only in damaged cells. Interestingly, these effects were only observed in wildtype MEFs, but not in *Inca1^−/−^* MEFs, indicating that INCA1 plays a key role in the regulation of S-phase progression by ING5. However, whether these effects of ING5 on DNA replication and S-phase progression contribute to its tumor suppressor or oncogenic functions needs to be further studied.

In summary, the current study identified several interacting partners of the CDK inhibitor INCA1. Our data show that by interacting with INCA1, ING5 exerts tumor-suppressive function by inhibiting colony formation and cell proliferation, and increasing Fas-antibody-induced apoptosis. However, without INCA1, ING5 no longer has anti-proliferative effects, and even functions as an oncogene as indicated by promoting cell proliferation and protecting cells from Fas-antibody-induced apoptosis. Mechanisms underlying the different behaviors of ING5 may include alternate splicing, post-translational protein modifications and protein-protein interactions that occur in different cellular contexts. Future study of ING5 should address these questions.

## Materials and Methods

### Yeast two-hybrid System

To identify INCA1-interacting proteins, a yeast two-hybrid (Y2H) screen was performed using the Matchmaker™ Gal4 two-hybrid system 3 (Clontech). All experiments were carried out according to the recommendations of the supplier (Clontech, Yeast Protocols Handbook, Matchmaker™ two-hybrid system 3). In brief, a human bone marrow cDNA library was cloned into the pGADT7 vector (Clontech). INCA1 served as bait for the library translation products. Full length human INCA1-cDNA was cloned into pGBKT7. The plasmids were co-transformed into the yeast strain AH109 (Clontech). Transformed yeast cells were grown on high stringency selection plates (-Leu, -Trp, -His, -Ade) with 5-bromo-4-chloro-3-indolyl-D-galactopyranoside (X-gal). Positive clones were further confirmed by colony-lift filter assay. Yeast plasmids were isolated and the cDNA inserts were confirmed by digestion with restriction endonucleases. Sequences were analyzed by alignment to the NCBI data bases and 30 genes from the positive clones were selected for further investigation.

### Patient samples, RNA isolation and qRT-PCR

Patient samples were collected from the bone marrow of AML patients. Written informed consent was obtained from all individuals. The use of the human material for scientific purposes was approved by the human ethics committees of the participating institution. The Ethics comitee full name is: “Ethik-Kommission der Ärztekammer Westfalen-Lippe und der medizinischen Fakultät der Westfälischen Wilhelms Universität Münster”.

Total RNA was isolated from 10 patients with normal bone marrow and 59 AML patients at the time of diagnosis using TRIzol reagent (Invitrogen, Carlsbad, CA). RNA (1 µg) was reverse-transcribed using random primers and MMLV reverse transcriptase (Promega, Madison, WI) following the manufacturer's protocol. ING5 mRNA expression levels were analyzed by real-time quantitative RT-PCR using TaqMan methodology as described [21]. Expression of the housekeeping gene glyceraldehyde-3-phosphate-dehydrogenase (GAPDH) was used to normalize the amount of cDNA between different testis samples [30]. ING5 primer and probe sequences will be provided on request.

### Cell Culture and Transfection

Murine Embryonic Fibroblasts (MEF) were established by trypsinating E12.5 embryos derived from breeding of *Inca1^+/−^* females and males [22]. Immortalization of fibroblasts was achieved with the standard 3T3 protocol. COS-7 (simian renal cells transformed by SV40, ECACC 87021302) cells and murine embryonic fibroblast (MEF) cells from *Inca1^+/+^* and *Inca1^−/−^* mice were cultured at 37°C and 5% CO_2_ in Dulbecco's modified Eagle's medium (Invitrogen) supplemented with 10% fetal calf serum (Biochrom KG, Berlin, Germany), 100 units/ml penicillin, and 100 µg/ml streptomycin (Biochrom) and 2 mM L-glutamine (Biochrom). Immortalization of MEFs was obtained following the standard 3T3 protocol. *Inca1* was cloned into pcDNA3.1(+) fused to enhanced green fluorescent protein (EGFP) (Clontech) as described previously [21]. pcDNA3.1-ING5 was a kind gift from Dr. Harris (Laboratory of Human Carcinogenesis, Center for Cancer Research, National Cancer Institute). COS-7 cells were transfected using SuperFect™ (QIAgen, Hilden, Germany) according to the manufacturer's protocol, and were used for co-immunoprecipitation. MEF cells were infected by retroviral particles (Pinco Expression Vector System; Pharmingen, San Diego, CA) with Lipofectamine Plus reagent (Invitrogen) according to the manufacturer's instructions.

### GST Fusion Proteins and GST Pull-down Assays

*Inca1* was cloned in frame into the pGEX-5X-2 plasmid (Amersham Biosciences) as GST fusion proteins, expressed in *Escherichia coli* BL21-DE3, and purified using glutathione-agarose beads, as described previously [21]. For GST pull-down assays, the screened gene sequences were expressed and radioactively labeled with [^35^S]methionine in an *in vitro* transcribed and translated TNT QuickCoupled Transcription/Translation System (Promega, Madison, WI), and then were incubated with INCA1 GST-proteins for 1 h at 4°C. After washing with binding buffer and SDS-PAGE, the gel was dried and analyzed by autoradiography.

### Antibodies, Western blotting and Co-immunoprecipitation

Radioimmune precipitation lysates (150 mM NaCl, 1% Nonidet P-40, 0.5% deoxycholic acid, 0.1% SDS, 50 mM Tris-HCl (pH 8.0)) with Complete EDTA-free protease inhibitor) or the indicated protein solutions were run on SDS-PAGE gradient gels (Bio-Rad). Subsequently, proteins were electroblotted onto PVDF membranes Immobilon-P (Millipore Corp., Bedford, MA), stained with specific primary antibodies and peroxidase-linked secondary antibodies (Jackson Immunoresearch Laboratories, West Grove, PA) and detected with ECL plus (Amersham Biosciences). Primary antibodies used for Western blot or immunoprecipitation were anti-actin (Sigma), anti-EGFP (Clontech), and anti-ING5 (Rockland Immunochemicals, Inc. PA). For co-immunoprecipitation, COS-7 cells were co-transfected with expression plasmids of EGFP-INCA1 and ING5. The cell lysates were precipitated with anti-ING5 or anti-EGFP antibodies, the bound proteins were subjected to SDS-PAGE and blotted for EGFP-INCA1 or ING5, as described previously [21].

### Colony Formation Assays

Colony formation assays of primary bone marrows obtained from *Inca1^+/+^* and *Inca1^−/−^* littermates were carried out essentially as described [31]. All animal experiments were performed with permission of the local veterinary administration (G15/2005 and 8.87-51.04.20.09-322). The animal welfare comitee/ageny is: “Landesamt für Natur, Umwelt und Verbraucherschutz NRW”.

For retroviral transduction, the packaging cell line Plat-E was transfected with the empty vector Pinco or pinco-ING5, and supernatants were collected every 12 hrs, starting 36 hrs after the transfection and the viruses were bound to retronectin-coated plates by centrifugation. Lineage-depleted bone marrow cells were growth-factor-stimulated overnight, transduced by growth on the virus-coated retronectin plates there times for 24 hrs and sorted by FACS for GFP-positivity. 1000 GFP-positive cells per milliliter methylcellulose were plated. Colony-forming units (CFU) were counted on day 7.

### Establishment of stable cell lines

Immortalized MEF cells from *Inca1^+/+^* and *Inca1^−/−^* mice were transduced with empty pinco vector or pinco-ING5. GFP-positive cells were sorted by FACS to establish ING5 overexpression MEF cells lines (*Inca1^+/+^*-ING5 and *Inca1^−/−^*-ING5) and control cells lines (*Inca1^+/+^*-control and *Inca1^−/−^*-control ).

### Promoter Activity and Luciferase Assay

For analyses of INCA1 and ING5 transactivation effects on the p53-responsive promoter, the consensus sequence of p53-binding sites was cloned into the pGL3 basic vector 5′ of a minimal TK promoter position to the firefly luciferase gene. Luciferase assays for promoter activity were carried out essentially as described [21] using the Dual-Luciferase reporter assay system (Promega, Madison, WI). A *Renilla* luciferase plasmid driven by a SV40 promoter and the luciferase reporter and expression construct were used to co-transfect *Inca1^−/−^* MEFs with expression vectors containing ING5, INCA1 or both. The promoter activity was determined as the ratio of firefly luciferase luminescence divided by *Renilla* luciferase activity. All experiments were carried out independently for three times, and data are indicated as mean with S.E.

### Proliferation assay

MEF cells were seeded into 96 well plates and cultured in 10% FCS or 0.1% FCS medium. [^3^H]-thymidine was incorporated overnight before cells were lysed. The proliferation rate was determined as described previously [21]. Results are presented from three independent experiments.

### Cell cycle Analysis

Cells were pulsed by BrdU for 1 hour before cells were collected by trypsinization at indicated time points. Samples were fixed in 70% ethanol at 4°C overnight. FITC labeled anti-BrdU and propidium iodide (PI, Sigma) staining was performed. DNA content was analyzed by flow cytometry with FACScan (Becton Dickinson, Mountain View, CA, USA) using the CELLQuest program (Becton Dickinson). Cell cycle distribution was analyzed by WinMDI software. Data presented are from three independent experiments.

### Apoptosis

Cells were treated with TNFα (200 ng/ml), TRAIL (100 ng/ml) or anti-Fas antibody (1 µg/ml) for 24 h or 48 h. Both adherent and non-adherent cells were harvested. The experiment was performed using the Annexin V-FITC Apoptosis Detection Kit (BD Pharmingen, USA) according to the manual.

### Statistical analysis

Data are shown as means plus SD from three independent experiments if not stable otherwise. Statistical comparisons were made using students' *t*-test. *P*<0.05 was considered statistically significant.

## Supporting Information

Figure S1 **Overexpression of ING5 decreased both membrane and total Fas expression.** The expression of membrane Fas and total Fas was detected by flow cytometry. ING5 overexpression down-regulated both the membrane and total Fas expression in *Inca1^+/+^* MEFs and *Inca1^−/−^* MEF cells. Data are shown as mean plus standard error of three independent experiments (**P<0.01 compared to control; *P<0.05 compared to control).
(TIF)
