# Supplementary figures and images for: The Inhibitor of Growth Protein 5 (ING5) Depends on INCA1 as a Co-Factor for Its Antiproliferative Effects
Source: PLoS One. 2011 Jul 5;6(7):e21505. doi: 10.1371/journal.pone.0021505 (PMC3130024; doi:10.1371/journal.pone.0021505)

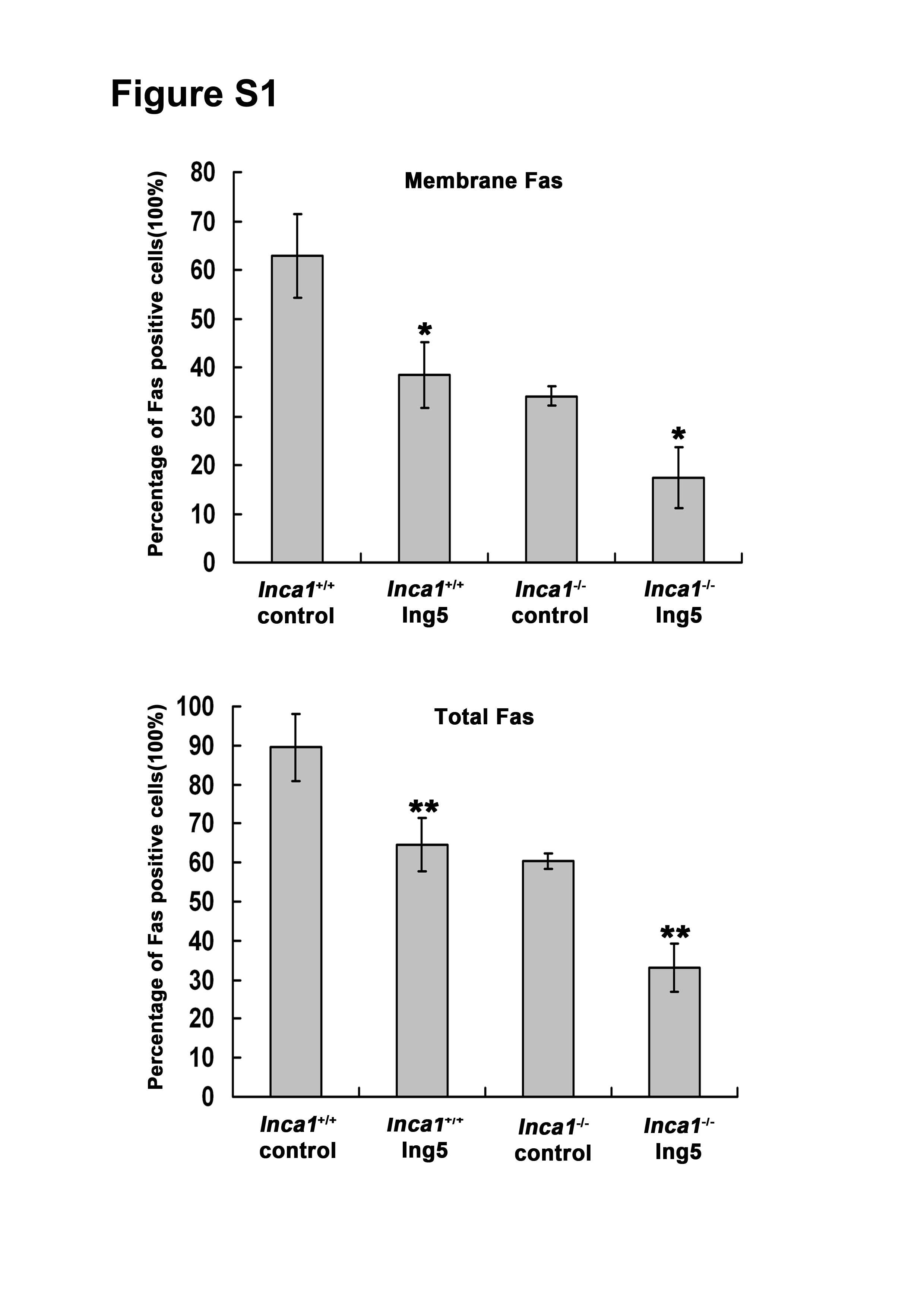

Supplement: Figure S1 — Overexpression of ING5 decreased both membrane and total Fas expression. The expression of membrane Fas and total Fas was detected by flow cytometry. ING5 overexpression down-regulated both the membrane and total Fas expression in Inca1+/+ MEFs and Inca1−/− MEF cells. Data are shown as mean plus standard error of three independent experiments (**P<0.01 compared to control; *P<0.05 compared to control). (TIF) [file pone.0021505.s001.tif]
